# Transcriptional Repressor Tbx3 Is Required for the Hormone-Sensing Cell Lineage in Mammary Epithelium

**DOI:** 10.1371/journal.pone.0110191

**Published:** 2014-10-24

**Authors:** Kamini Kunasegaran, Victor Ho, Ted H-. T. Chang, Duvini De Silva, Martijn L. Bakker, Vincent M. Christoffels, Alexandra M. Pietersen

**Affiliations:** 1 Department of Cellular and Molecular Research, National Cancer Centre Singapore, Singapore, Singapore; 2 Program in Cancer & Stem Cell Biology, Duke-NUS Graduate Medical School Singapore, Singapore, Singapore; 3 Center for Heart Failure Research, Academic Medical Centre, Amsterdam, The Netherlands; 4 Department of Physiology, National University of Singapore, Singapore, Singapore; Baylor College of Medicine, United States of America

## Abstract

The transcriptional repressor Tbx3 is involved in lineage specification in several tissues during embryonic development. Germ-line mutations in the Tbx3 gene give rise to Ulnar-Mammary Syndrome (comprising reduced breast development) and Tbx3 is required for mammary epithelial cell identity in the embryo. Notably Tbx3 has been implicated in breast cancer, which develops in adult mammary epithelium, but the role of Tbx3 in distinct cell types of the adult mammary gland has not yet been characterized. Using a fluorescent reporter knock-in mouse, we show that in adult virgin mice Tbx3 is highly expressed in luminal cells that express hormone receptors, and not in luminal cells of the alveolar lineage (cells primed for milk production). Flow cytometry identified Tbx3 expression already in progenitor cells of the hormone-sensing lineage and co-immunofluorescence confirmed a strict correlation between estrogen receptor (ER) and Tbx3 expression in situ. Using in vivo reconstitution assays we demonstrate that Tbx3 is functionally relevant for this lineage because knockdown of Tbx3 in primary mammary epithelial cells prevented the formation of ER+ cells, but not luminal ER- or basal cells. Interestingly, genes that are repressed by Tbx3 in other cell types, such as E-cadherin, are not repressed in hormone-sensing cells, highlighting that transcriptional targets of Tbx3 are cell type specific. In summary, we provide the first analysis of Tbx3 expression in the adult mammary gland at a single cell level and show that Tbx3 is important for the generation of hormone-sensing cells.

## Introduction

Tbx3 is a transcriptional repressor with an important role in embryonic development of the mammary gland and a high expression in certain breast cancers, but its role in the different cell types of adult mammary epithelium has yet to be explored [1]. Mammary gland development starts in the embryo, but the largest part occurs postnatally. During murine embryogenesis, an ectodermal mammary placode is induced which develops into a rudimentary epithelial tree [2]. During puberty, under the influence of steroid hormones, the epithelial ducts start to elongate and bifurcate to fill the mammary fat pad [3]. In the adult, morphogenesis of the mammary gland continues as it is subject to further branching and the development of lobular structures with alveoli (milk-producing units) during pregnancy, culminating in lactation, followed by regression and remodelling to a virgin-like state after weaning. At a smaller scale, there is even some alveologenesis and regression under the influence of hormonal fluctuations during the estrus cycle [3].

Milk ducts in the adult virgin are bi-layered with a luminal layer that consists of hormone-sensing cells and cells primed for milk production (alveolar progenitor cells) and an outer basal layer that contains mostly contractile myoepithelial cells, but also rare mammary epithelial stem cells [4]. Both these multipotent stem cells as well as lineage-restricted populations contribute to epithelial renewal and alveologenesis [5]–[7]. In transplantation assays, a progenitor that gives rise to all cells types of an alveolus can be detected [8], but recent data by several groups [7], [9], [10] highlights that in intact mammary glands alveoli are commonly formed by collaborative outgrowth of cells from at least 3 distinct lineages. This includes cells from the basal lineage, the luminal estrogen receptor-negative (ER-) alveolar lineage and the luminal ER+ hormone-sensing lineage [10]. The latter was unexpected, since hormone-sensing cells have been considered mature, or terminally differentiated cells. However several reports have shown that hormone-sensing cells actively proliferate in vivo in early pregnancy [11], [12]. In addition, ER+ progenitor cells have recently been identified by cell surface markers and in vitro colony forming potential [13], [14], indicating that it is indeed a separate lineage. The regulation of the hormone-sensing lineage is particularly interesting because the majority of breast cancers express the estrogen receptor [15], [16].

Here, we analyzed the role of Tbx3 in the lineage hierarchy of the adult mammary gland. Tbx3 is one of the earliest markers of mammary epithelial cells in embryonic development, and in the absence of Tbx3 embryonic mammary placodes fail to form [17]. In Tbx3-heterozygote mice, reduced expression of Tbx3 is sufficient to allow normal mammary gland development [17], although a later study showed that in thoracic mammary glands epithelial trees occasionally did not form and fewer branches were observed in the adult glands [18]. In humans, hypomorphic germline mutations in the Tbx3 gene are the cause of Ulnar-Mammary Syndrome [19] in which reduced activity of Tbx3 results in reduced breast development, in addition to other developmental defects [20]. Thus, even though there appear to be differential quantitative requirements for Tbx3, Tbx3 plays an important role in early mammary gland development across species. Tbx3 is also involved in the embryonic development of numerous other tissues, including limbs, heart and liver [21], [22].

Tbx3 is likely to play a role in adult tissues as well, because Tbx3 has been implicated as an oncogene [23], [24] and was found overexpressed in cell lines from several cancer types, including melanoma and hepatoma [24], [25]. In breast cancer, high nuclear and cytoplasmic expression of Tbx3 was found in a subset of cells in primary tumors [26] and high transcriptional Tbx3 levels in human breast tumors correlated strongly with ER expression [27]. Here, we investigated the expression of Tbx3 in the different mammary epithelial cell types in the adult mammary gland.

## Materials and Methods

### Ethics statement

Mice used in this study were maintained under protocols which were approved by the legal authority of the Singhealth Institutional Animal Care and Use Committee, Singapore. All procedures were in accordance with its guidelines. Non-terminal procedures were performed under anesthesia, and all efforts were made to minimize suffering of the animals. Anesthesia (Hypnorm:Midazolam:Water  =  1∶1∶2) was administered at 7 ml/kg and analgesia (Meloxicam) was administered at 0.2 mg/kg. Further analgesia (Meloxicam) was added to the drinking water for 2–3 days after surgery (∼0.2 mg/kg). Animals were euthanized by carbon dioxide inhalation followed by cervical dislocation. All efforts were taken to prevent animal suffering.

### Mice

Tbx3^tm1(Venus)Vmc^ (synonym: Tbx3^Venus^) mice were generated by inserting the Venus coding and transcription termination/pA sequences into the start codon of Tbx3. This places Venus under control of the endogenous Tbx3 locus and prevents expression of Tbx3 itself. A detailed description of the generation of these knock-in mice is in preparation and will be described elsewhere (MLB and VMC). Heterozygous Tbx3^+/Venus^ mice were maintained on an FVB background. Mice were euthanized by carbon dioxide inhalation and immediately dissected for thoracic (MG3), abdominal (MG4 & 5) mammary glands. Animal care and protocols were in accordance with national and institutional guidelines.

### Cell labeling, flow cytometric analysis & fluorescence-activated cell sorting (FACS)

Abdominal glands were pooled and processed for single mammary epithelial cell (MEC) isolation for FACS analysis as previously described [28]. Fluorochrome-conjugated antibodies were titrated on primary mammary epithelial cells to ensure maximal positive signal:background fluorescence ratio. Anti-mouse &/or anti-rat compensation beads (BD 552843 and 552845, respectively) were used for single stain antibody controls. Compensation controls also included two cellular samples: unstained cells and cells stained with DAPI (Sigma D8417, USA). Cells were incubated with antibodies on ice for 45 minutes with agitation every 15 minutes. Samples were then washed with twice the sample volume and resuspended in L15 (Gibco-Life Technologies, USA) with 6% FCS (Hyclone, USA) and 200 ng/mL of DAPI, except non-DAPI compensation controls. All multiple-labelled samples were gated on FSC-A vs. SSC-A and doublet discrimination (FSC-H vs. FSC-W & SSC-H vs. SSC-W) and DAPI negativity. Samples contained anti-CD45 to exclude lymphocytes from analysis. Cells were analyzed and sorted on a BD FACS-Aria II containing 355 nm UV, 488 nm blue, 561 nm yellow-green and 633 nm red lasers. Specific antibodies used and gating strategy are detailed in File S1.

### Synthesis of cDNA & qPCR analysis

For analysis of transcript levels by qPCR in FACS sorted populations, cells were sorted directly into lysis buffer (10 IU RNase inhibitor (Invitrogen, USA), 2 mM DTT, 0.15% Tween-20 (Biorad, USA) in 12 µL of nuclease-free water) in PCR tubes using a Direct Reverse Transcription method as described [29]. Five-hundred cells were sorted into each tube and Reverse transcription (RT) was performed using Superscript VILO (Invitrogen, USA) as per manufacturers protocol. Primers were designed that span introns to exclude the detection of genomic DNA and selected for optimum melt curve and amplification profiles (for primer sequences, see File S2). qPCR was performed using Sso Fast Evagreen supermix reagent (Biorad #172–500) as per manufacturers protocol.

For microarray analysis, MECs from 3 Tbx3^+/Venus^ adult virgin mice were pooled and 200,000 luminal Venus^High^ and luminal Venus^Low^ cells were sorted into L15 medium with 6% FCS. After centrifugation, cell pellets were lysed in Trizol and total RNA was isolated according to manufacturers protocol. Biotinylated cRNA was prepared from 250 ng of total RNA with the GeneChip 3′ IVT Express Kit according to the manufacturers protocol (Affymetrix 2008). Following fragmentation, 12.5 ug of cRNA was hybridized on a GeneChip Mouse Genome 430 2.0 Array for 16 hours at 45 C. The GeneChip was washed and stained in the GeneChip Fluidics Station 450 (Affymetrix) and scanned using the GeneChip Scanner 3000 7 G (Affymetrix). A log 2 base transformation was applied before the data was normalized. Normalization and centering of all genes for each sample was performed by BRB Array software. The data is deposited as GEO series GSE58327.

### Indirect immunofluorescence

Thoracic mammary glands were fixed for 24 hours in 4% paraformaldehyde and embedded in paraffin wax. Paraffin sections of 5 µm were prepared and subjected to 1 mM disodium-EDTA antigen retrieval as described previously [28]. Primary antibodies used for immunofluorescence are the following: Cytokeratin 8 (Developmental Studies Hybridoma Bank TROMA-I, rat, 1∶100), Estrogen receptor (Novocastra NCL-ER-6F11, mouse, 1∶100), E-Cadherin (BD Biosciences 610181, mouse, 1∶250), Progesterone receptor (Abnova MAB9785, rabbit, 1∶400), Smooth muscle actin (Sigma A2547, mouse, 1∶1000), Tbx3 (Invitrogen 424800, rabbit, 1∶100), turboGFP (Pierce Antibodies PA522688, rabbit, 1∶400), turboGFP (OriGene TA150041, mouse 1∶250). Secondary antibodies used at 1∶400 dilution are from Invitrogen: Alexa488-coupled goat anti-mouse (A11029), Alexa488-coupled goat anti-rabbit (A11034), Alexa568-coupled goat anti-mouse (A11031) and Alexa568-coupled goat anti-rabbit (A11036). Additionally, CF633nm-coupled donkey anti-rat (Biotium 20137-1) was used at 1∶400 dilution. Images were acquired on a Zeiss LSM-710 confocal microscope with a pinhole aperture of 1 Airy unit.

### Colony forming assays

For analysis of colony forming potential, a pool of 4–6 Tbx3^+/Venus^ mice between the age of 10 to 16 weeks were used. One thousand sorted cells were seeded into 22.1 mm (12-well) plate with 0.75−1×10^6^ NIH-3T3 feeder cells that had been treated with mytomycin C (10 ug/ml) (Sigma, M4287). Colonies were cultured for 5–6 days in MEC medium, consisting of DMEM:F12 medium (Invitrogen, 11320033) supplemented with Penicillin Streptomycin (Gibco, 15140), 6% FCS (Hyclone, SV30160.03), 5 ng/ml cholera toxin (Sigma, 8052), 5 ug/ml Insulin (Sigma, 16634) and 10 ng/ml EGF (Sigma, E4127). At the end of the assay colonies were fixed in 4% paraformaldehyde and stained with 1 mg/ml crystal violet (Sigma, C3886) in 50% dH_2_0: 50% methanol. Images were acquired on a Olympus IX71 inverted microscope.

### Lentiviral vector construct & production

Several short hairpins against murine Tbx3 were cloned into a MSCV-blast vector using a miR30 backbone and tested for knock down efficiency in HC11 cells. The two best shRNAs (see File S2) were digested with SalI and MluI and ligated into the GIPZ cloning vector (Trans-Lentiviral Packaging kit TLP4616, Thermo Scientific Open Biosystems) digested with XhoI and MluI (New England Biolabs). Lentiviral particles were produced by co-transfecting 32 ug of GIPZ plasmid (non-silencing or with a shRNA against Tbx3), 10 ug of pSuper-Drosha (to reduce processing of the RNA during packaging) and 30 ug packaging plasmids (TLP mix) into HEK293T (HCL4517, Thermo Scientific Open Biosystems) using the calcium phosphate method according to the manufacturers' protocol.

### Transplantation

Abdominal mammary glands were harvested from 5 wildtype FVB (experiment 1) and 4 KI (Tbx3^+/Venus^) donor mice (experiment 2). The mammary glands were digested to single cells and plated at 5×10^5^ cells/well in a 6-well plate. Cells were adhered overnight in 3% oxygen and the next day cells were subjected to spin transduction. Viral supernatant was diluted in MEC medium at a 2∶1 ratio, added to the cells at 1.5 ml/well and spun at 2000 rpm for 30 minutes at 32°C. After centrifugation, the cells were returned to the incubator (5% CO2 and 3% O2). The next morning, cells were washed thrice with PBS and trypsinized, spun and re-suspended in 10–20 µl MEC medium. Cells from one well were injected into cleared MG4 fat pads of 21-day old matched recipient mice and allowed to engraft for 10 weeks [30]. Glands were then harvested, fixed in methacarn (60% methanol, 30% chloroform, 10% acetic acid) or 4% paraformaldehyde for 24 hours and embedded in paraffin.

## Results

### Tbx3 is differentially expressed in mammary epithelial cellular subsets

**Figure 1:**
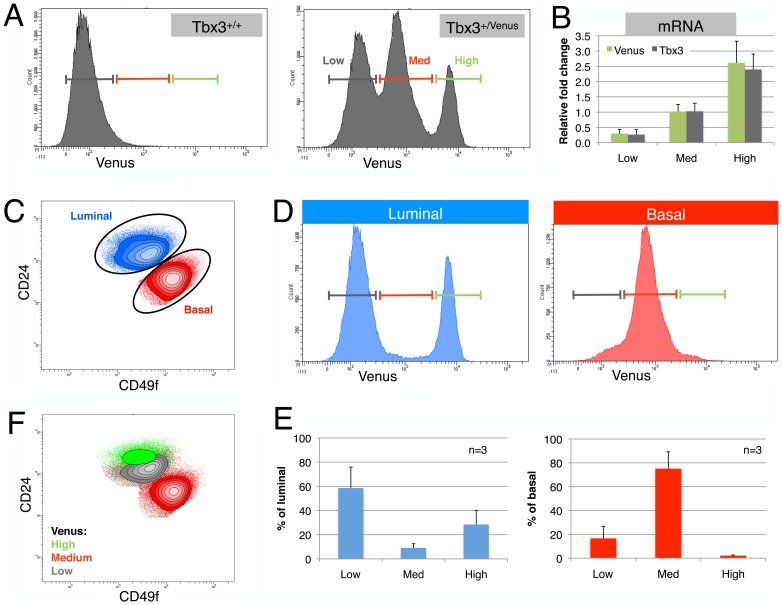
Fluorescent reporter reveals distinct Tbx3 expression in mammary epithelial cell subsets. (A) Epithelial cells isolated from mammary glands of wildtype (Tbx3^+/+^) or knock-in (Tbx3^+/Venus^) mice show three peaks with different levels of Venus expression (Low, Medium and High). (B) Mammary epithelial cells (MECs) from 3 independent Tbx3^+/Venus^ animals were sorted according to Venus signal intensity. qPCR on 500 directly lysed cells shows that both Venus and Tbx3 mRNA correlates tightly with Venus fluorescence intensity. (C) MECs were labeled with fluorescent antibodies against CD24 and α6-integrin (CD49f) to distinguish the luminal (blue) and basal (red) cell populations. (D) When plotted separately, luminal cells from Tbx3^+/Venus^ mammary glands show two main populations; Venus^Low^ (cells with low Tbx3 expression) and Venus^High^ (cells with high Tbx3 expression). Basal cells express intermediate level of Venus (and Tbx3). (E) Quantification of the percentage of Venus-Low, -Medium and -High cells in the luminal and in the basal population of Tbx3^+/Venus^ epithelium. Data are presented as mean ± SD of three individual adult virgin Tbx3^+/Venus^ animals. (F) Populations gated based on Tbx3 expression (Venus-Low, -Medium and -High) plotted on a CD24/CD49f contour plot.

To investigate Tbx3 expression in the different mammary epithelial cell (MEC) types, we made use of a reporter mouse strain in which one of the Tbx3 alleles had been replaced with the gene for Venus, a variant of yellow fluorescent protein (Tbx3^+/Venus^, also referred to as knock-in (KI) mice). We isolated primary MECs from Tbx3 wildtype and KI females and analyzed them by flow cytometry. After excluding dead cells, lymphocytes and stromal cells (see gating strategy in File S1), the epithelial cells segregated in three distinct peaks based on Venus signal intensity (Figure 1A). Venus expression accurately reflected Tbx3 transcription in the three different peaks, with the highest Tbx3 mRNA levels in the cells with the highest Venus fluorescence (Figure 1B).

To identify the cell type with high Tbx3 expression, we separated MECs into cells of the luminal layer (CD24^hi^CD49f^lo^, blue) and basal layer (CD24^l^°CD49f^hi^, red, Figure 1C). When the luminal and basal cellular subsets are plotted separately, it is apparent that the peak with intermediate Venus signal reflected basal cells, and that luminal cells were divided into a subset with high and one with low Tbx3 promoter activity (Figure 1D). This distribution is quantified in independent animals in Figure 1E. Plotting the populations with distinct Venus intensities on a CD24/CD49f contour plot further illustrates the division of the luminal population based on Tbx3 expression (Figure 1F).

### High Tbx3 expression in hormone-sensing cells

**Figure 2:**
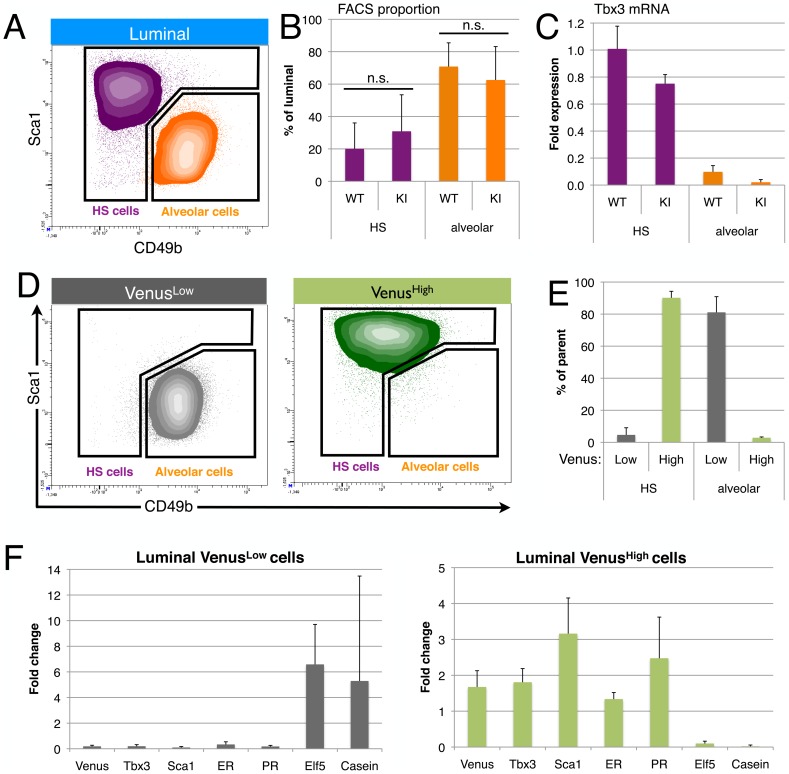
Tbx3 marks hormone sensing cells. (A) Luminal cells from wildtype mammary glands are separated into hormone-sensing (HS, Sca1^hi^CD49b^lo^, purple) and alveolar (Sca1^l^°CD49b^hi^, orange) subsets based on Sca1 and alpha2-integrin (CD49b) expression. (B) There is no significant (n.s.) difference in the proportion of hormone-sensing (HS, purple) and alveolar cells (orange) between Tbx3^+/+^ (wildtype, WT) and Tbx3^+/Venus^ (Knock-in, KI), paired t-test p = 0.53 for HS and p = 0.60 for Alv. (C) Tbx3 mRNA levels in sorted populations as indicated. (D) Tbx3^+/Venus^ luminal cells were first gated for Low or High Venus expression (see Figure 1D), and then plotted based on Sca1 and CD49b expression. (E) Proportion of hormone-sensing (HS, purple) and alveolar cells (orange) that are Venus^Low^ (grey) or Venus^High^ (green), measured by FACS in 3 independent Tbx3^+/Venus^ animals. (F) Fold change in mRNA expression in luminal Venus^Low^ (left panel) or Venus^High^ (right panel) cells, relative to total luminal population. Data are presented as mean ± SD of three adult virgin Tbx3^+/Venus^ animals.

Epithelial cells in the luminal layer fall into two main functional categories, hormone-sensing (HS) cells and alveolar progenitor cells and these can be separated by flow cytometry using the additional cell surface markers Sca1 and CD49b (Figure 2A). The proportion of HS and alveolar progenitor cells in Tbx3^+/Venus^ mammary epithelium is similar to that in wildtype litter mates (Figure 2B), indicating that Tbx3 heterozygosity does not affect the composition of the luminal layer. This could be the result of relatively high Tbx3 mRNA levels in the heterozygote KI cells (75% of wildtype cells, Figure 2C), which might suggest that Tbx3 is involved in a negative transcriptional feedback loop. This experiment also demonstrated that Tbx3 expression is highest in the HS population (Figure 2C), raising the question whether the cells with highest Tbx3 expression are all hormone-sensing cells. Plotting luminal cells based on their Tbx3 expression showed that indeed almost all Venus^High^ cells were part of the HS cell population whereas almost all Venus^Low^ cells belonged to the alveolar progenitor cell population (Figure 2D). Similarly, separating the luminal population based on cell type also showed that the majority of the HS cell population was Venus^High^ and the alveolar progenitor population was Venus^Low^ (Figure 2E). The correlation between high Tbx3 expression and a hormone-sensing cell identity was confirmed by transcriptional analysis by microarray using cells pooled from three animals and separated by Venus fluorescence (File S3) and by qPCR on luminal populations sorted from individual KI animals (Figure 2F). Luminal cells with low levels of Venus and Tbx3 expressed variable but high levels of Elf5, a transcription factor that specifies alveolar cell fate [31], and beta-Casein, one of the components of milk (Figure 2E). Luminal cells that expressed high levels of Tbx3 had high levels of Sca1 transcription, in line with the flow cytometry profiles, and expressed high levels of the estrogen and progesterone receptor, thereby confirming the hormone-sensing identity of Tbx3-expressing luminal cells at the molecular level.

### Tbx3 expression marks the hormone-sensing cell lineage

**Figure 3:**
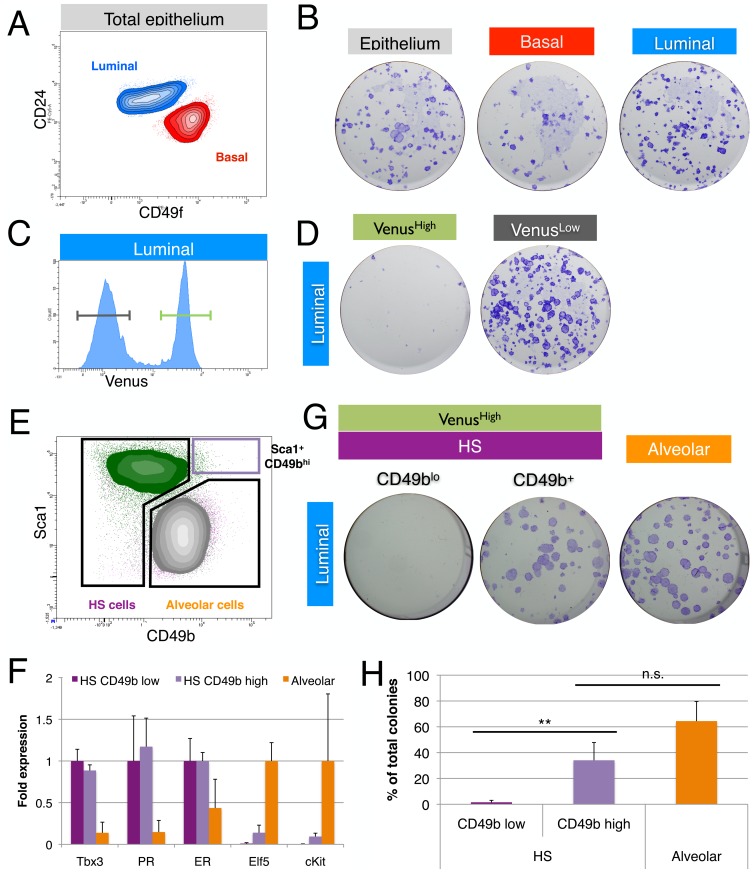
Tbx3 marks the hormone sensing lineage, including ER+ progenitor cells. (A) Combined density/contour plot of mammary epithelial cells (from a pool of 5 Tbx3^+/Venus^ mice) separated into basal (red) and luminal (blue) cells based on CD24 and alpha6-integrin (CD49f) expression. (B) Colony forming potential of 1000 sorted cells from each population, representative of two independent experiments. (C) Histogram of luminal mammary epithelial cells Venus^High^ (luminal) and Venus^Low^ (luminal) cells sorted for colony forming assay (from a pool of 5 Tbx3^+/Venus^ mice per experiment). (D) Colony forming potential of 1000 sorted cells from each population, representative of two independent experiments. (E) FACS profile of hormone-sensing (CD49b^high^ and CD49b^low^) and alveolar progenitor cells that were used for colony assays. Cells are color-coded based on Venus expression (green = Venus^High^, grey = Venus^Low^). (F) Fold change in Tbx3, progesterone receptor (PR) and estrogen receptor (ER) mRNA expression of sorted CD49b^high^ and CD49b^low^ hormone-sensing cells and alveolar progenitor cells, relative to CD49b^low^ hormone-sensing cells (dark purple bar). Fold change in Elf5 and cKit mRNA expression is shown relative to luminal alveolar cells (orange bar). (G) Colony forming potential of 1000 sorted luminal cells: CD49b^low^ and CD49b^high^ hormone-sensing cells and alveolar progenitor cells. (H) Quantification of colony forming assays with HS cells (Sca1^high^CD49b^low^), HS progenitor cells (Sca1^high^Cd49b^high^) and alveolar progenitor cells (Sca1^low^CD49b^high^). Bars represent the mean of three independent pools of 5–6 adult virgin Tbx3^+/Venus^ animals ± SD. HS progenitor cells form more colonies than HS cells (p = 0.02, paired t-test) and there is no significant difference in colony forming potential between HS progenitor and alveolar progenitor cells (p = 0.21, paired t-test).

In the non-pregnant adult mammary gland, proliferation is detected mostly in ER- luminal cells, and this is mirrored by their colony forming potential when plated on feeder layers [13], [32]. Similarly, when we sorted mammary epithelial cells from adult virgin mice, we found that luminal cells form more colonies than basal cells (Figure 3A, 3B). The luminal colony-forming potential was derived almost entirely from Tbx3-Venus^Low^ cells (Figure 3C, 3D) as expected based on their alveolar ER- cell identity. Luminal cells with high Tbx3 expression lacked colony-forming potential (Figure 3D), consistent with other reports that show that the HS cell population is non-clonogenic [13], [33]. Nevertheless, Shehata and colleagues recently identified a small subset of hormone-sensing progenitor cells that expressed high levels of alpha2-integrin (Sca1^hi^CD49b^hi^) and formed colonies on feeder cells [13]. Analyzing this ER+ progenitor population separately demonstrated that the majority of these cells belonged to the Tbx3-Venus^High^ population (94%±3%, Figure 3E) and mRNA levels of both Tbx3 and hormone receptors in Sca1^hi^CD49b^hi^ cells were comparable to that of the general hormone-sensing cell population (Figure 3F). In line with previous studies [13], [14], the ER+ progenitor population is distinct in that it also expressed low levels of Elf5 and c-Kit. Luminal cells with high Tbx3 expression (Venus^High^) that fell into the Sca1^hi^CD49b^hi^ gate had robust colony forming potential (Figure 3G, quantified in Figure 3H), confirming the progenitor potential of this population. These experiments showed that Tbx3 expression is already high in the earliest recognizable cell type of the hormone-sensing lineage.

Taken together, analysis of the Tbx3^+/Venus^ reporter by flow cytometry combined with functional assays demonstrates that in the luminal layer of mammary epithelium Tbx3 expression distinguishes the hormone-sensing cell lineage from the secretory alveolar lineage.

### Tbx3 protein expression in intact mammary glands

**Figure 4:**
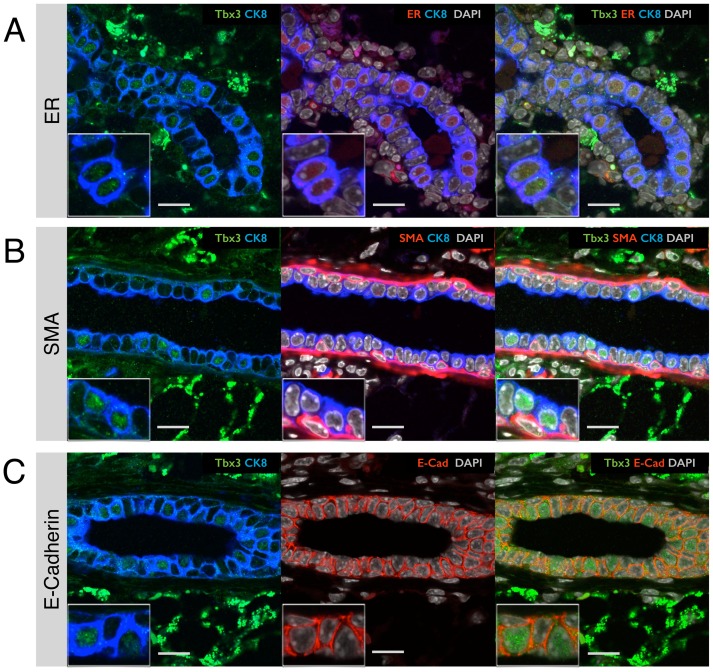
Tbx3 expression in epithelial cells in intact mammary glands. (A–C) Confocal immunofluorescence on paraffin sections of mammary glands from wildtype adult virgin mice. (A) Ductal structure probed with antibodies for Tbx3 (green), the estrogen receptor (ER, red) and the luminal cell marker cytokeratin-8 (CK8, blue). Nuclei are stained with DAPI (grey). (B) Duct probed for Tbx3 (green), the basal marker smooth muscle actin (SMA, red) and CK8 (blue). (C) Duct probed for Tbx3 (green), E-Cadherin (E-cad, red) and CK8 (blue). Images are representative of staining performed on paraffin sections of 3 independent animals. Scale bar is 20 µm or 10 µm for the inset.

To further evaluate whether the strict correlation between transcriptional expression of both Tbx3 and ER that we observed by FACS holds true at the protein level in intact mammary glands, we used co-immunofluorescence on paraffin sections from adult virgin mice. The Tbx3 antibody gave high background staining in the stroma, but clearly confirmed the existence of cells in the luminal layer of the epithelial ducts with either a strong or undetectable signal for Tbx3 in the nucleus (Figure 4A). The ER+ cells were 99.7%±0.25% Tbx3 positive, with only 4 out of 1391 ER+ cells lacking Tbx3 expression. We did not observe any Tbx3-positive cells that did not express ER in adult virgin mice.

At the transcriptional level, basal cells displayed intermediate Tbx3 expression (Figure 1D). On paraffin sections, it was difficult to detect Tbx3 protein in cells of the basal layer (identified by the basal marker smooth muscle actin, see Figure 4B). This could be due to post-transcriptional regulation by for instance microRNAs, but it could also be a technical limitation of the sensitivity of detection. In the luminal layer, cells strongly express E-cadherin, including the hormone-sensing cells with high Tbx3 expression (Figure 4C). Tbx3 was found to repress E-cadherin in melanoma cells [24], but it does not appear to do so in mammary epithelial cells.

**Figure 5:**
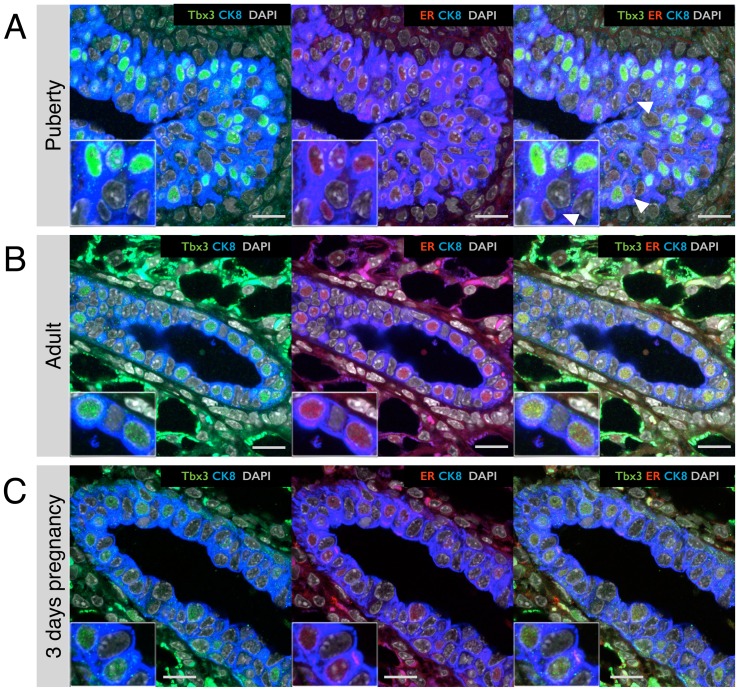
Correlation between Tbx3 and ER expression at different developmental stages of postnatal mammary gland development. Confocal immunofluorescence staining of Tbx3 (green), ER (red) and luminal marker cytokeratin-8 (CK8, blue) on mammary glands from (A) 5-week old pubertal mice (terminal end bud structure), (B) 10-week old virgin mice (ductal structure) and (C) 3 day pregnant mice (ductal structure). Nuclei are stained with DAPI (grey). White arrow heads indicate cells in terminal end bud (A) with ER expression but no Tbx3 expression. Images are representative of staining performed on paraffin sections of 3 independent animals. Scale bar is 20 µm.

Previous studies have shown that Tbx3 is involved in development of the rudimentary mammary gland during embryogenesis [17], a process that is steroid hormone independent [3]. During puberty, steroid hormones induce the elongation of mammary epithelial ducts. The invasive tips of elongating milk ducts are called Terminal End Buds (TEBs) and we examined the pattern of Tbx3 expression in these structures using mammary glands from pubertal mice. Similar to the adult mammary gland, the majority of ER+ cells in TEBs expressed Tbx3 (92.2%±2.2%, Figure 5A). However in puberty the occurrence of ER+ cells without Tbx3 (2.6%±3.4%) was more prevalent compared to the adult virgin, and in puberty there were some Tbx3-positive cells without detectable ER expression (4.3%±1.7%). The staining for Tbx3 appeared more intense in TEBs compared to quiescent adult mammary tissue (Figure 5B). In puberty, a considerable proportion of ER+ cells is proliferating [34], in contrast to the quiescent adult stage. In early pregnancy, ER+ cells also proliferate [11] but Tbx3 levels did not seem elevated during this stage of active morphogenesis (Figure 5C). At day 3 of pregnancy, there appeared to be a near perfect correlation between ER and Tbx3, similar to the adult virgin. However, due to a lower staining intensity for both ER and Tbx3 at this stage putative cells that express only one or the other might be more difficult to detect.

Together, these experiments show that Tbx3 expression consistently distinguishes hormone-sensing cells from ER- luminal cells, not only by flow cytometry but also in unperturbed mammary tissue at different developmental stages.

### Tbx3 is required for the hormone-sensing cell lineage

**Figure 6:**
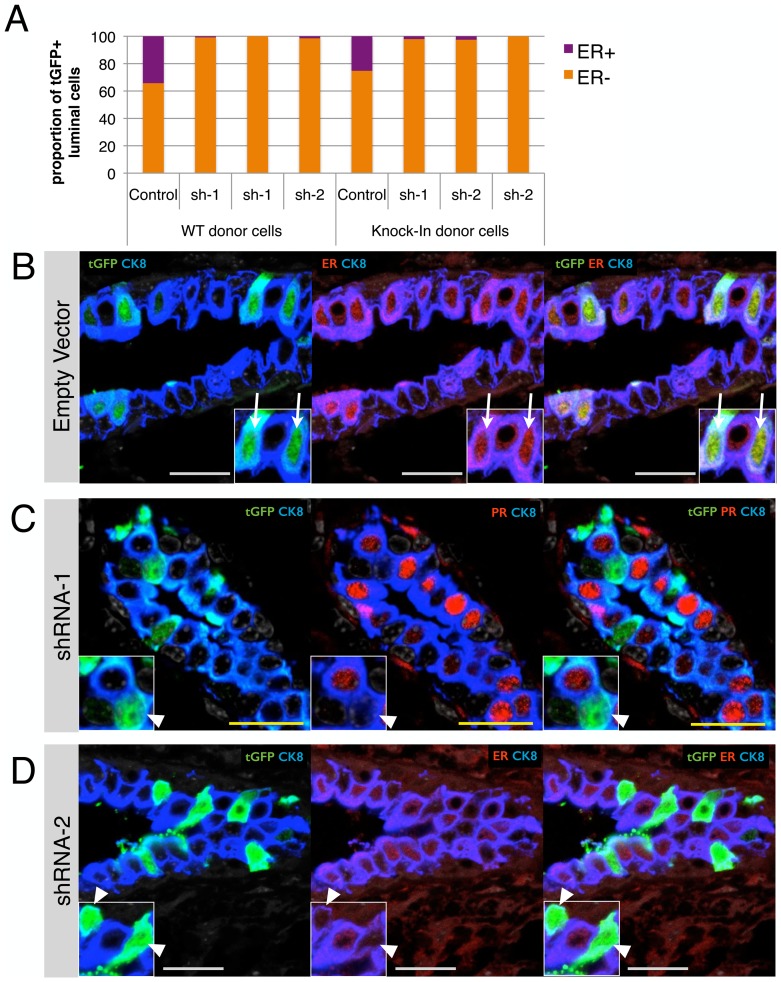
Tbx3 is required for the generation of hormone-sensing cells. (A) Primary MECs from wildtype or KI mice were transduced with a non-silencing lentiviral vector (control) or with two independent short hairpins against Tbx3 (sh-1 and sh-2). Cells were injected into mammary fat pads devoid of endogenous epithelium and outgrowths were analyzed 8–10 weeks later for the identity of lentivirally transduced cells (recognized by tGFP expression). See File S4B for a schematic experimental design. Each bar represents one fat pad and 46 to 569 tGFP+ luminal cells were counted per fat pad. There is a significant bias against the formation of HS cells by cells with Tbx3 knockdown (Chi square of shRNA versus control transplant <0.01). (B) Paraffin section of a cleared mammary fat pad transplanted with MECs that were exposed to the non-silencing control vector. Transduced cells are identified with an antibody staining against tGFP (green), luminal cells are identified by cytokeratin 8 (blue) and HS cells are identified by the estrogen receptor (ER, red). White arrow indicates transduced cells contributing to the hormone-sensing lineage. (C) Paraffin section of a mammary fat pad transplanted with MECs exposed to the first short hairpin against Tbx3. White arrow head indicated transduced cells in the luminal alveolar (ER-negative) lineage. The background of immunohistochemistry is higher in transplanted samples (arguably due to fibrosis). Where ER staining was ambiguous due to high background, we used progesterone receptor (PR, red) staining as an alternative marker for HS cells. (D) Paraffin section of a mammary fat pad transplanted with MECs exposed to the second short hairpin against Tbx3. White arrow head indicates transduced cells in the luminal alveolar (ER-negative) lineage. White scale bar is 20 µm and yellow scale bar is 10 µm.

To determine if Tbx3 expression is functionally relevant for the hormone-sensing cell lineage, we designed two short hairpins that target distinct regions of the murine Tbx3 transcript. The reduction in Tbx3 mRNA by the shRNAs was confirmed in a mouse mammary epithelial cell line (HC11) after selection of lentivirally-transduced cells by puromycin (File S4A). Next, freshly isolated MECs were incubated with lentiviral vectors overnight and transplanted (without selection) into mammary fat pads devoid of endogenous epithelium (see File S4B for the experimental design). Eight to ten weeks after reconstitution, we examined the identity of the cells that were transduced by lentiviral vectors (as indicated by their turboGFP (tGFP) expression). Outgrowths from cells that had been exposed to the control virus illustrated that the majority of transduced cells were lineage-restricted progenitors; we rarely found ducts whereby all cell types were tGFP positive. Instead, we found ducts that contained tGFP+ cells that predominantly belonged to one lineage (either luminal ER- or ER+ or basal, see File S5). For these experiments, we used two different pools of donor cells; one consisting of wildtype MECs and the other consisting of Tbx3^+/Venus^ MECs to potentially facilitate further knockdown. Importantly, in both cases the pool of cells transduced with the control vector gave rise to cells of all different lineages, including ER+ hormone-sensing cells (Figure 6A&B). Cells with Tbx3 knockdown robustly gave rise to luminal ER- cells, but there was a strong bias against the generation of hormone-sensing cells (Figure 6C&D). This was true for both the wildtype and KI cells and for both short hairpins targeting Tbx3 (Figure 6A). In both control and Tbx3 knockdown outgrowths we found some tGFP+ cells that contributed to cells of the basal layer but this proportion was too low for accurate quantification. The lack of adverse effects of Tbx3 knockdown in the Tbx3^Low^ alveolar lineage and the strong effect in the Tbx3-positive hormone-sensing lineage demonstrate that Tbx3 expression is important for the generation of hormone-sensing cells.

## Discussion

Tbx3 is required for mammary epithelial cell identity early in embryogenesis, but its role in the different mammary epithelial cell types of the adult mammary gland had not yet been determined. Using a novel reporter mouse strain, we show that Tbx3 transcriptional expression is tightly regulated at different levels in the three main epithelial lineages; high in hormone-sensing cells, very low in alveolar progenitor cells and intermediate in basal cells.

Based on the striking bimodal distribution of Tbx3 in the luminal population, we focused our analysis in this study on the role of Tbx3 in luminal mammary epithelial cells. During embryonic heart and liver development, Tbx3 plays an important role in specific lineage choices. For instance, in embryonic heart development, Tbx3 is involved in a lineage choice between pacemaker cells and atrial cardiac cells, in which Tbx3 represses atrial genes in pacemaker cells [35], [36]. Ectopic expression of Tbx3 can impose a pacemaker phenotype on atrial cells [36], showing that in this context Tbx3 can direct cell fate.

Adult mammary epithelium is actively renewed by stem cells and lineage-restricted progenitors [5]. The bimodal expression of Tbx3 in the luminal lineage, together with the expression of Tbx3 in ER+ progenitors, suggested that Tbx3 may play a role in the lineage choice between the hormone-sensing and the alveolar lineage. Indeed, we found that knockdown of Tbx3 in primary mammary epithelial cells strongly reduced the formation of ER+ cells in mammary reconstitution assays, demonstrating that Tbx3 is required for a hormone-sensing cell fate. We have tried to express a Tbx3 transgene in alveolar progenitor cells to determine if Tbx3 could impose a hormone-sensing cell identity, but we found only few cells that expressed Tbx3 ectopically *in vivo* (data not shown). This could be due to an anti-proliferative effect of Tbx3 [37], however ectopic Tbx3 expression *in vitro* did not prevent proliferation of ER- cells. Therefore, we cannot rule out that the scarcity of Tbx3 overexpressing cells *in vivo* was due to a technical limitation.

The differential expression of Tbx3 in the luminal lineage raises the question of which signaling pathways influence Tbx3 transcription. Based on other studies using cell lines combined with our observations presented in this study, we can speculate what signals are most likely involved in regulating Tbx3 expression in the intact adult mammary gland. For instance, in ER+ breast cancer cell lines, Tbx3 expression was dependent on both estrogen and FGF signaling [27]. In primary tissue of murine mammary glands, we found a strong correlation between ER and Tbx3 expression and it is therefore plausible that in adult mammary epithelium Tbx3 is downstream of estrogen signaling. It was shown that FGF signaling is required for Tbx3 expression in mammary epithelial cells in the embryo [38]. At this time the three distinct adult lineages do not yet exist [6], [7] and mammary development is hormone independent [3]. FGF signaling is also active in pubertal mammary gland development [39], [40] and we observed strong Tbx3 staining in TEBs, raising the possibility that FGF signaling also contributes to Tbx3 expression in postnatal mammary epithelium. However, ER+ and ER- luminal cells derived from adult mammary glands both express FGF receptors and a downstream target of FGF signaling, Dusp6, is expressed even higher in ER- luminal cells (File S6), and it is therefore unlikely that FGF signaling is responsible for the hormone-sensing cell specific expression of Tbx3.

Another pathway that may contribute to Tbx3 expression is TGFβ signaling. In MCF12A cells, a non-transformed mammary epithelial cell line, TGFβ directly induced Tbx3 transcription [37]. Notably, TGFβ actively inhibits proliferation of MCF12A cells, and also prevents proliferation of ER+ cells in the mammary gland [41]. In MCF12A cells, Tbx3 was required for the anti-proliferative effects of TGFβ [37]. This is surprising because studies with other cell types suggested an oncogenic role of Tbx3. For instance, Tbx3 was found to directly bind and repress the tumor suppressor Arf in mouse embryonic fibroblasts [42], thus preventing activation of p53. However, Tbx3 might have other target genes in the mammary gland, since both Arf and p21 expression are robustly detectable on the microarray of sorted primary Tbx3-VenusHigh cells (File S6). Moreover, loss of Arf or p53 did not rescue developmental defects of mammary placodes in Tbx3-mutants [18]. Apart from Arf and p21 not many direct targets for Tbx3 have been described. Tbx3 targets in the mouse heart include genes for gap junctions and ion channels [43] and in embryonic stem cells Tbx3 binds promoters of genes that are also regulated by pluripotency factors [44], underscoring that Tbx3 likely regulates different target genes depending on the cellular context [21]. Indeed, genes that are repressed by Tbx3 such as E-cadherin in melanoma cells [24] and p21 in promoter assays [45], are highly expressed in Tbx3-positive mammary epithelial cells (Figure 4C and File S3). Determining the cistrome of Tbx3 specifically in hormone-sensing cells will likely help to unravel the role of Tbx3 in normal and malignant mammary gland development.

In primary human breast tissue obtained from reduction mammoplasties, Tbx3 expression is also highest in a population of luminal cells that expresses high levels of steroid receptors [13], indicating that Tbx3 is a marker for hormone-sensing cells in human mammary epithelium as well. Interestingly, this population is also predominantly non-clonogenic [13], which raises the possibility that Tbx3 has indeed an anti-proliferative role in hormone-sensing cells in normal breast tissue. This would be in line with data from sequencing breast cancer genomes; Tbx3 mutations are found specifically in ER+ breast cancers and these mutations are predicted to result in loss of function [46], [47]. This indicates that Tbx3 might actively prevent tumorigenesis of hormone-sensing cells resulting in selection pressure to lose that function. However, it is possible that in tumors that have retained wildtype Tbx3 expression, Tbx3 is involved in promoting migration [48] or paracrine stimulation of proliferation [27]. Considering these potential conflicting roles of Tbx3 in breast tumorigenesis, it will be important to further characterize the specific role of Tbx3 in breast tissue before considering inhibition of Tbx3 for cancer therapy [49].

In summary, we provide the first characterization of Tbx3 expression at the single cell level in the adult mammary gland. By different methods we demonstrate that Tbx3 is highly expressed in the hormone-sensing cell lineage and is functionally required for the generation of hormone-sensing cells during mammary morphogenesis. Our data highlight that Tbx3 is likely to regulate a distinct set of target genes depending on the cellular context. Given the current uncertainty about an anti- or pro-tumorigenic role of Tbx3 in breast cancer, it will be interesting to determine the relevant targets for Tbx3 specifically in hormone-sensing cells.

## Supporting Information

File S1 **FACS sorting of primary MECs.** Antibodies used in FACS sorting for separating the different mammary epithelial populations. (B) Gating strategy for FACS analysis and sorting.
(TIF)

File S2 **Primer sequences.** (A) Polymerase chain reaction (PCR) primers used for gene expression quantification by quantitative PCR (qPCR). (B) Target sequences for the short hairpins in Tbx3, and the short hairpin against Drosha used during viral vector production.
(PDF)

File S3 **Top 100 genes highest in luminal Venus High vs Low cells.** Tab 1. Top 100 genes highest expressed in luminal Venus^High^ cells compared to luminal Venus^Low^ cells based on Affymetrix microarray using cells pooled from three animals that were separated by Venus fluorescence. Several described markers for hormone-sensing cells are highlighted in bold. Tab 2. Top 100 genes highest expressed in luminal Venus^Low^ cells compared to luminal Venus^High^ cells. Several described markers for alveolar cells are highlighted in bold.
(XLSX)

File S4 **Transplantation of lentivirally-transduced MECs.** (A) mRNA levels of Tbx3 from puromycin-selected HC11 that were transduced with either empty vector or short hairpins targeting Tbx3. (B) Experimental set up for lentiviral transduction of MECs and subsequent transplantation into cleared mammary fat pads of 21-day old recipient mice. (C) For each condition a small aliquot of cells was plated on coverslips while the rest of the cells was used for transplantation. The cells on coverslips were analysed after 48 hours in culture to ensure similar transduction efficiency (visualized by tGFP expression) by the different lentiviral supernatants.
(TIF)

File S5 **Examples of transduced lineage-restricted progenitors.** Paraffin sections of mammary outgrowths of MECs transduced with lentiviral vectors. Transduced cells are identified with an antibody staining against tGFP (green), luminal cells are identified by cytokeratin 8 (blue) and HS cells are identified by the estrogen or progesterone receptor (ER or PR, red). (A) Example of an outgrowth containing transduced cells that belong to the luminal alveolar (ER-negative) lineage (tGFP+CK8+ER-, white arrow head). (B) Example of an outgrowth containing transduced cells that belong to the luminal hormone-sensing lineage (tGFP+CK8+PR+, white arrow). (C) Example of an outgrowth containing transduced cells that belong to the basal lineage (tGFP+CK8-ER-, white arrow head). (D) Transplanted fat pads were fixed with either paraformaldehyde (PFA) or methacarn. Representative images of secondary antibody control stainings on both types of fixed tissue are shown. Scale bar is 20 µm.
(TIF)

File S6 **Selected genes from microarray.** Expression of FGF receptors & ligands, cell cycle inhibitors and E-cadherin in luminal Venus^High^ and Venus^Low^ cells (Affymetrix log2 values).
(XLSX)
