# Supplementary figures and images for: Transcriptional Repressor Tbx3 Is Required for the Hormone-Sensing Cell Lineage in Mammary Epithelium
Source: PLoS One. 2014 Oct 24;9(10):e110191. doi: 10.1371/journal.pone.0110191 (PMC4208772; doi:10.1371/journal.pone.0110191)

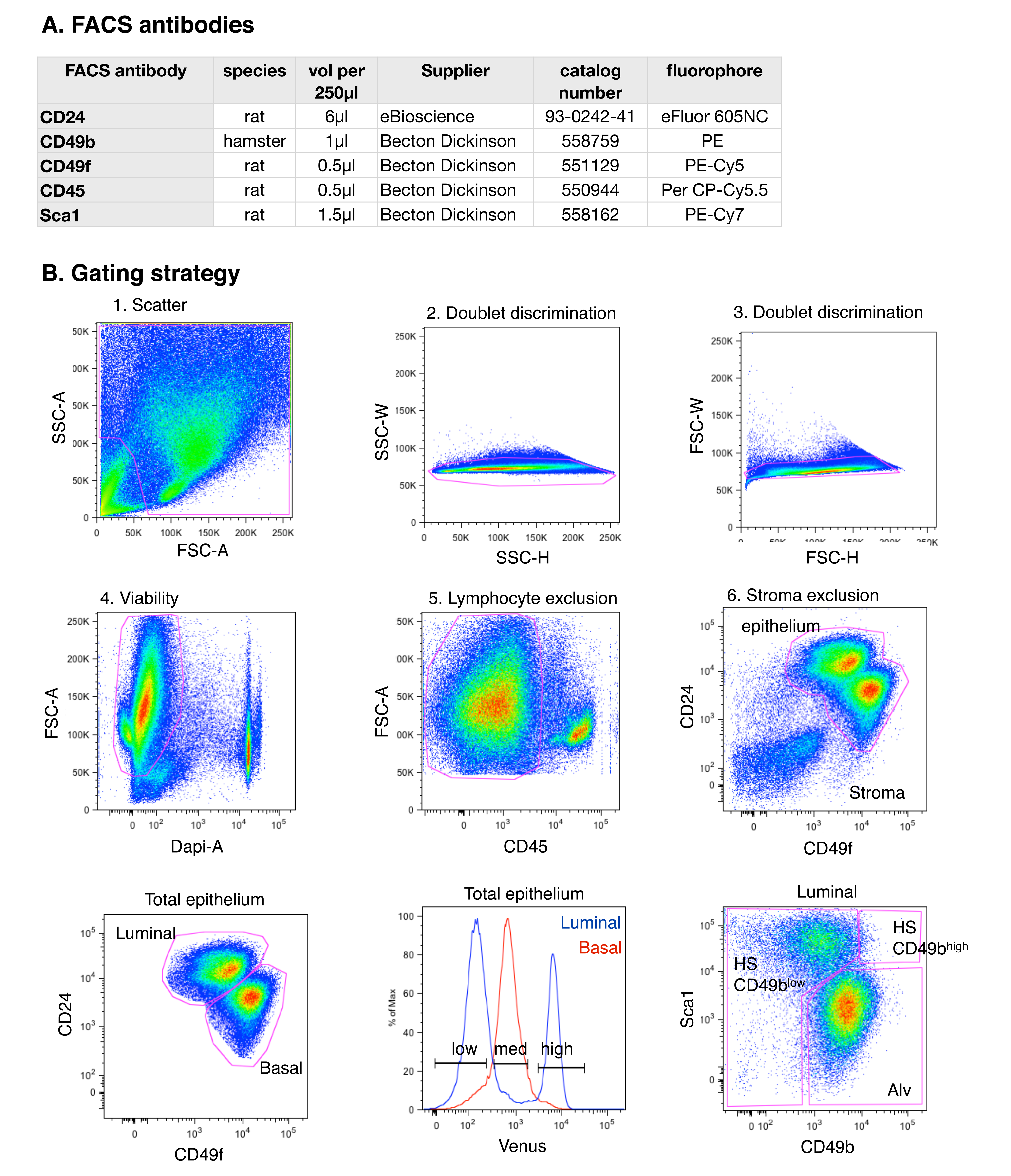

Supplement: File S1 — FACS sorting of primary MECs. Antibodies used in FACS sorting for separating the different mammary epithelial populations. (B) Gating strategy for FACS analysis and sorting. (TIF) [file pone.0110191.s001.tif]

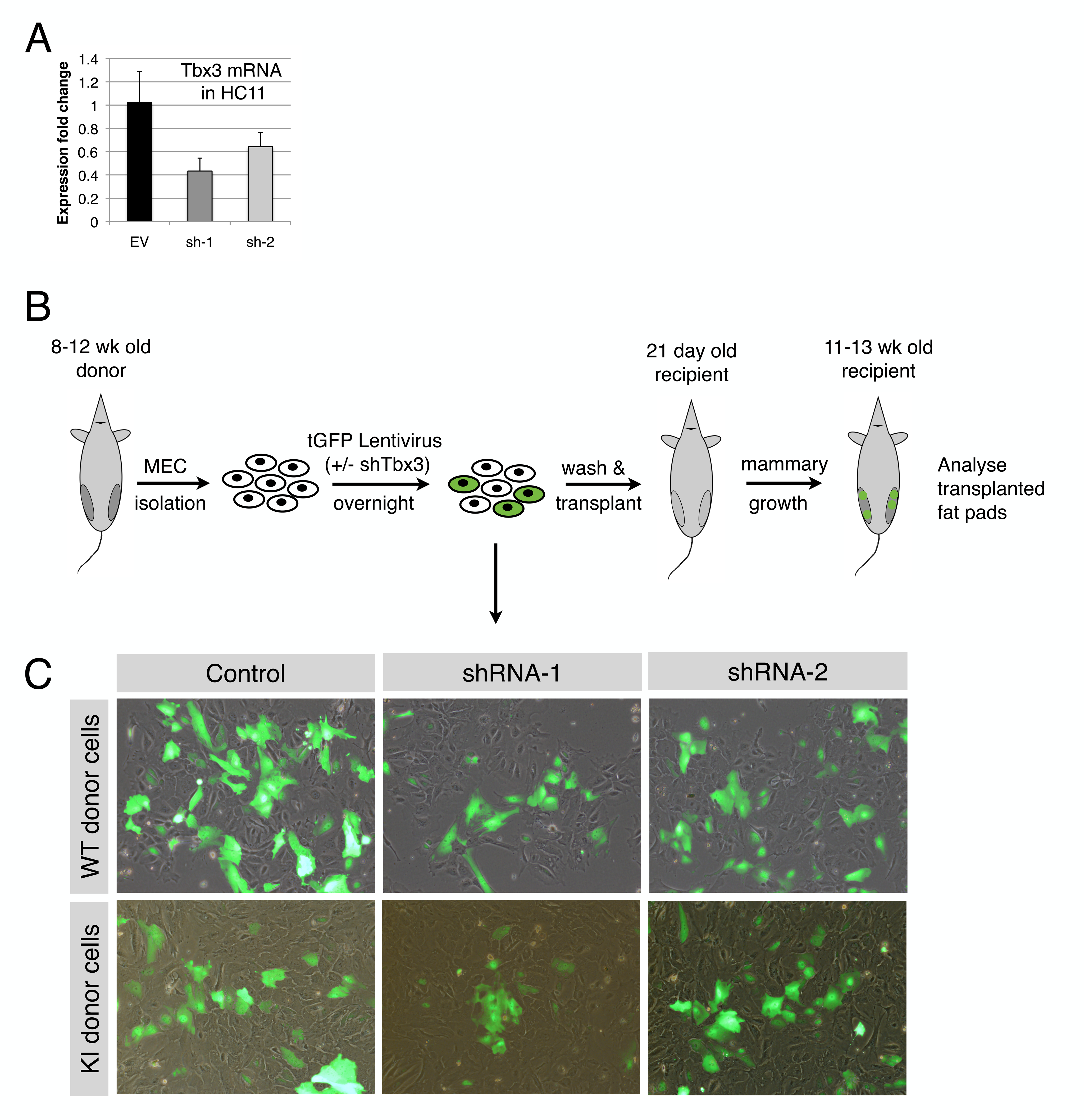

Supplement: File S4 — Transplantation of lentivirally-transduced MECs. (A) mRNA levels of Tbx3 from puromycin-selected HC11 that were transduced with either empty vector or short hairpins targeting Tbx3. (B) Experimental set up for lentiviral transduction of MECs and subsequent transplantation into cleared mammary fat pads of 21-day old recipient mice. (C) For each condition a small aliquot of cells was plated on coverslips while the rest of the cells was used for transplantation. The cells on coverslips were analysed after 48 hours in culture to ensure similar transduction efficiency (visualized by tGFP expression) by the different lentiviral supernatants. (TIF) [file pone.0110191.s004.tif]

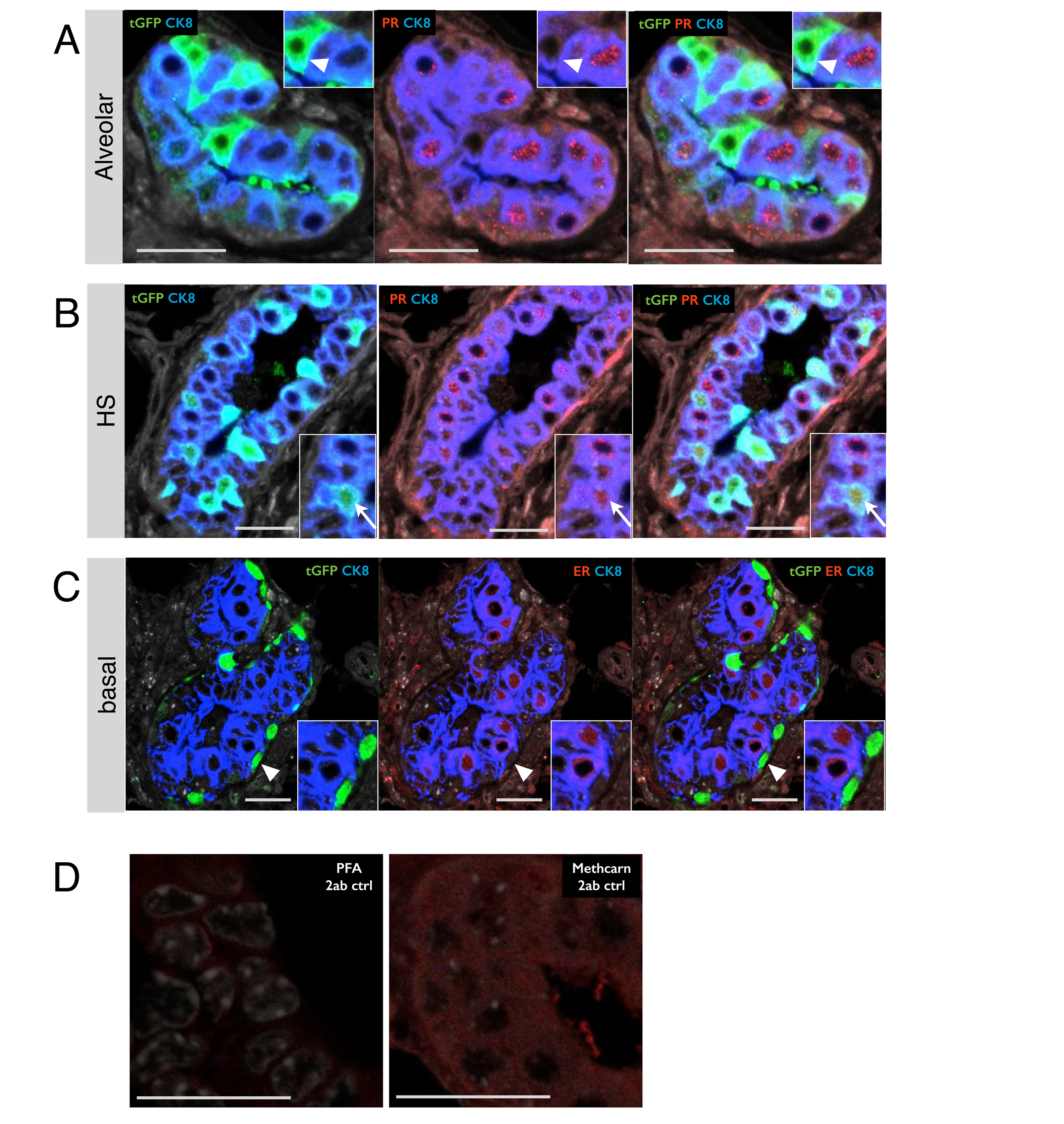

Supplement: File S5 — Examples of transduced lineage-restricted progenitors. Paraffin sections of mammary outgrowths of MECs transduced with lentiviral vectors. Transduced cells are identified with an antibody staining against tGFP (green), luminal cells are identified by cytokeratin 8 (blue) and HS cells are identified by the estrogen or progesterone receptor (ER or PR, red). (A) Example of an outgrowth containing transduced cells that belong to the luminal alveolar (ER-negative) lineage (tGFP+CK8+ER-, white arrow head). (B) Example of an outgrowth containing transduced cells that belong to the luminal hormone-sensing lineage (tGFP+CK8+PR+, white arrow). (C) Example of an outgrowth containing transduced cells that belong to the basal lineage (tGFP+CK8-ER-, white arrow head). (D) Transplanted fat pads were fixed with either paraformaldehyde (PFA) or methacarn. Representative images of secondary antibody control stainings on both types of fixed tissue are shown. Scale bar is 20 µm. (TIF) [file pone.0110191.s005.tif]
