# Supplementary material for: Transcriptional Repressor Tbx3 Is Required for the Hormone-Sensing Cell Lineage in Mammary Epithelium
Source: PLoS One. 2014 Oct 24;9(10):e110191. doi: 10.1371/journal.pone.0110191 (PMC4208772; doi:10.1371/journal.pone.0110191)
Supplement: File S2 — Primer sequences. (A) Polymerase chain reaction (PCR) primers used for gene expression quantification by quantitative PCR (qPCR). (B) Target sequences for the short hairpins in Tbx3, and the short hairpin against Drosha used during viral vector production. (PDF) [file pone.0110191.s002.pdf]

Supplemental File S2. primer sequences

A. qPCR primers

Gene	Forward primer	Reverse primer
Tbx3	TTGCAAAGGGTTTTCGAGAC	GTTGGATGTCCCCACGAT
GFP (and Venus)	CACATGAAGCAGCACGACTTCT	AACTCCAGCAGGACCATGTGAT
Sca1	TGCAACCTTGTCTGAGAGGA	TGGGACTCCATAGCACTGGT
Estrogen receptor alpha (ESR1)	GCCAAGGAGACTCGCTACTG	CTCCGGTTCTTGTCAATGGT
Progesterone receptor (PgR)	GGTGGAGGTCGTACAAGCAT	CTCATGGGTCACCTGGAGTT
Elf5	GGACTCCGTAACCCATAGCA	TACTGGTCGCAGCAGAATTG
β-Casein (Csn2)	TCCACAACATTCCGTTTCTG	AGCATGATCCAAAGGTGAAAA
HPRT	CTGGTGAAAAGGACCTCTCG	TGAAGTACTCATTATAGTCAAGGGCA
cKit	TCCTCTGGGAGCTCTTCTCCTT	GTTGGACAACCTGCTTGAATGTT

B. shRNA sequences

	Target sequence
shRNA-1 mouse Tbx3 (in ORF)	GCCACCCGTTCCTCAATTTGAACAGCA
shRNA-2 mouse Tbx3 (in 3' UTR)	CCCTTCGTGTGTTTAGTGGAAACATCCCC
shRNA Drosha in pSuper	GGACCAAGTATTCAGCAAG
